# Comparing Selections of Environmental Variables for Ecological Studies: A Focus on Terrain Attributes

**DOI:** 10.1371/journal.pone.0167128

**Published:** 2016-12-21

**Authors:** Vincent Lecours, Craig J. Brown, Rodolphe Devillers, Vanessa L. Lucieer, Evan N. Edinger

**Affiliations:** 1 Department of Geography, Memorial University of Newfoundland, 232 Elizabeth Avenue, St. John’s, Newfoundland and Labrador, Canada; 2 Applied Research, Nova Scotia Community College, 80 Mawiomi Place, Dartmouth, Nova Scotia, Canada; 3 Institute for Marine and Antarctic Studies, University of Tasmania, 20 Castray Esplanade, Battery Point, Tasmania, Australia; 4 Department of Biology, Memorial University of Newfoundland, 232 Elizabeth Avenue, St. John’s, Newfoundland and Labrador, Canada; Universitat Trier, GERMANY

## Abstract

Selecting appropriate environmental variables is a key step in ecology. Terrain attributes (*e*.*g*. slope, rugosity) are routinely used as abiotic surrogates of species distribution and to produce habitat maps that can be used in decision-making for conservation or management. Selecting appropriate terrain attributes for ecological studies may be a challenging process that can lead users to select a subjective, potentially sub-optimal combination of attributes for their applications. The objective of this paper is to assess the impacts of subjectively selecting terrain attributes for ecological applications by comparing the performance of different combinations of terrain attributes in the production of habitat maps and species distribution models. Seven different selections of terrain attributes, alone or in combination with other environmental variables, were used to map benthic habitats of German Bank (off Nova Scotia, Canada). 29 maps of potential habitats based on unsupervised classifications of biophysical characteristics of German Bank were produced, and 29 species distribution models of sea scallops were generated using MaxEnt. The performances of the 58 maps were quantified and compared to evaluate the effectiveness of the various combinations of environmental variables. One of the combinations of terrain attributes–recommended in a related study and that includes a measure of relative position, slope, two measures of orientation, topographic mean and a measure of rugosity–yielded better results than the other selections for both methodologies, confirming that they together best describe terrain properties. Important differences in performance (up to 47% in accuracy measurement) and spatial outputs (up to 58% in spatial distribution of habitats) highlighted the importance of carefully selecting variables for ecological applications. This paper demonstrates that making a subjective choice of variables may reduce map accuracy and produce maps that do not adequately represent habitats and species distributions, thus having important implications when these maps are used for decision-making.

## Introduction

Due to the difficulty in sampling ecological data at sufficient spatial and temporal resolutions, many ecological studies rely on the use of surrogates to understand species distribution and ecological processes. Amongst commonly used surrogates, terrain attributes (*e*.*g*. slope, rugosity, aspect) derived from digital elevation (DEM) or bathymetric (DBM) models have proven their value in a broad range of terrestrial and marine ecological studies [1]. Such attributes can now be derived easily using tools available in most Geographic Information Systems (GIS). While tools are increasingly user-friendly, a lack of transparency in most software on the actual algorithms used [2] can prevent users from making an informed decision on which tools to use. Also, terrain attributes sharing the same name but generated using different algorithms (*e*.*g*. slope) have been shown to produce different derivative surfaces [2,3]. Software developers and authors of published work are often not explicit on the methods they use to derive terrain attributes (*e*.*g*. algorithm or tool). This lack of information can possibly influence the analysis and interpretation of the resulting terrain attribute surfaces, and consequently the ecological application for which they are being used.

Choosing an appropriate selection of terrain attributes for specific ecological applications can be challenging, and users will often simply use the terrain attributes made available by the software they have access to or are familiar with, without further questioning if those attributes are the most appropriate ones for their study. In a related study, Lecours *et al*. [4] showed that many terrain attributes covary, which may cause potential problems for many statistical analyses. In a seabed classification context, Diesing *et al*. [5] recommended integrating the reduction of covariation within practices. In an attempt to identify an optimal combination of terrain attributes to use in ecology that would reduce covariation while extracting as much information as possible on the terrain, Lecours *et al*. [4] recommended using a combination of six easily computable terrain attributes for ecological studies that consider topography or bathymetry: (1) relative deviation from mean value, which is a measure of relative position that can identify local peaks and valleys, (2) standard deviation, which is a measure of rugosity, (3) local mean, (4) slope, and (5–6) easterness and northerness, which together provide information on the orientation of the slope (*i*.*e*. aspect).

This article aims to describe the effects of subjectively selecting input variables for ecological applications, with a particular focus on terrain attributes. The specific objectives are (1) to compare the performance of Lecours *et al*. [4] recommended selection of terrain attributes to other selections in a real ecological context, (2) to demonstrate the relative importance of terrain morphology in aiding our understanding of ecological questions compared to other environmental variables, and (3) to report on the consequences of selecting different input variables on both the accuracy of habitat maps and the spatial distribution of the outputs.

## Materials and Methods

Benthic habitat mapping is the act of mapping significantly distinct areas of the seafloor based on their physical, chemical and biological characteristics at particular spatial and temporal scales [6]. The marine environment presents particular challenges in observing and sampling seafloor characteristics. However, developments in acoustic remote sensing technologies, specifically multibeam echosounders (MBES), now allow the collection of high-resolution remotely sensed data of the seafloor. Bathymetric measurements from MBES can be used to generate DBMs, from which terrain attributes can be derived [7]. Additionally, MBES systems can also record acoustic reflectance (backscatter) data that provide information on seafloor properties (*e*.*g*. surficial geology, porosity). In combination, these attributes are commonly used for the production of benthic habitat maps. For the purpose of this study, two common approaches to habitat mapping were used: unsupervised and supervised classifications.

### Data

**Fig 1:**
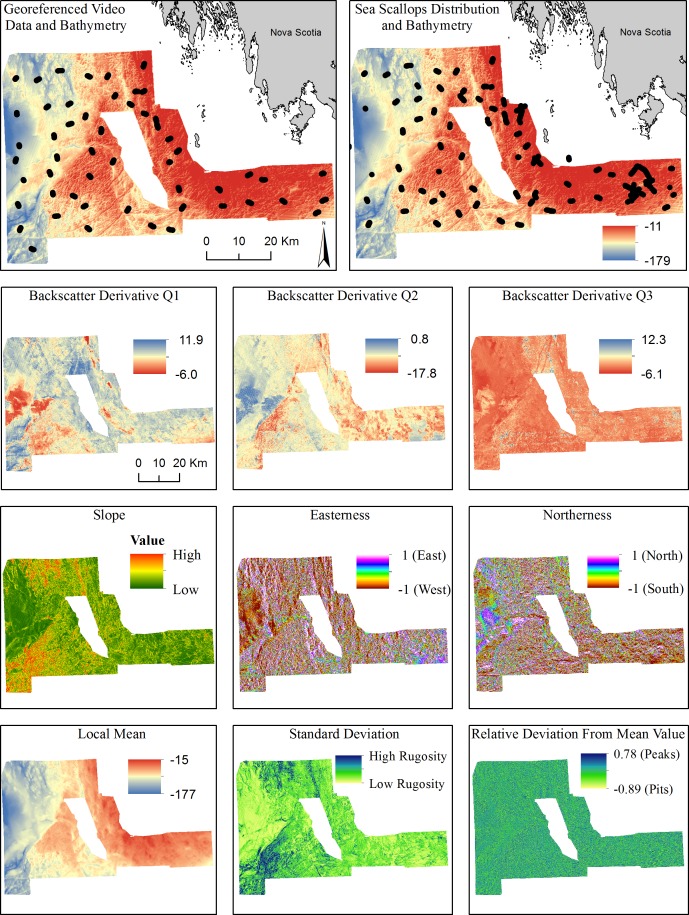
German Bank study area with some of the input variables used in this study: the ground-truth data for the bottom types, the sea scallops observations, the bathymetry, the three backscatter derivatives and the six terrain attributes from Selection 1.

Datasets from Brown *et al*. [8], covering 3,650 km^2^ of German Bank, an area of the Canadian continental shelf off Nova Scotia (Fig 1), were used to address the objectives of this study. These data comprised a 50 m resolution DBM, 3,190 geo-referenced underwater images of the seabed visually classified into five bottom types (glacial till, silt and mud, rippled silt, rippled sand, reef), 4,816 geo-referenced sea scallop observations, and three backscatter data derivatives (Q1, Q2, Q3; Fig 1). Details on how the data were collected and processed can be found in Brown *et al*. [8]. For comparison with surfaces used in Lecours *et al*. [4], the fractal dimension, which is a quantitative representation of surface complexity, was measured over 10,000 m^2^ areas of German Bank. Values ranged from 2.09 to 2.93, thus including regions of low (towards 2.00), moderate and high complexities (towards 3.00).

A total of 24 different terrain attributes were derived from the DBM and grouped into seven selections of six terrain attributes each (Table 1). The terrain attributes were selected from groups of variables that exhibited various behaviours during the statistical analyses performed in Lecours *et al*. [4] (see caption of Table 1 and S1 Appendix for more details). Selection 1 corresponds to our recommended selection of six terrain attributes. These terrain attributes were computed using the TASSE (Terrain Attribute Selection for Spatial Ecology) toolbox for ArcGIS [9]. Selections 2 to 7 were built to maximize variability and resemblance to Selection 1 (*e*.*g*.to avoid having two measures of slope or curvatures within one selection). Particular focus was also given to terrain attributes that were identified as potentially important by Lecours *et al*. [4].

**Table 1:** Selections of terrain attributes used to build the habitat maps and models. The ID numbers refer to Lecours *et al*. [4] and allow finding the software and parameters with which the attributes were generated (see also S1 Appendix). Marker variables correspond to important variables; whether they were found on strong components (Sel. 1) or weak components (Sel. 4) is linked to the amount of topographic structure they accounted for. Variables with low cardinality (Sel. 2) did not have many different values, thus limiting their ability to explain slight variations in terrain morphology. Complex variables (Sel. 3) correspond to redundant variables. The terrain attributes identified by an asterisk were previously identified as potentially important [4]. The underlined attributes were recommended in [4].

**Selection 1**		**Selection 2**		**Selection 3**		**Selection 4**	
*Marker Variables on Strong Components*		*Variables with Low Cardinality*		*Complex Variables*		*Marker Variables on Weak Components*	
ID31	Easterness	ID1	Bathymetric Position Index	ID70	Mean of Residuals*	ID132	Plan Curvature
ID67	Local Mean	ID2	Center vs Neighbor Variability*	ID116	Plan Curvature	ID153	Profile Curvature
ID90	Northerness	ID42	Easterness*	ID136	Profile Curvature	ID158	Representativeness*
ID157	Relative Deviation from Mean Value	ID101	Northerness*	ID178	Slope	ID188	Slope Variability
ID166	Slope	ID111	Percentile	ID201	Surface Roughness	ID219	Total Curvature
ID190	Standard Deviation	ID143	Profile Curvature	ID221	Value Range	ID227	Vector Ruggedness Index
**Selection 5**		**Selection 6**		**Selection 7**	
*Mix of Selections 1 and 2*		*Mix of Selections 1 and 3*		*Mix of Selections 1 and 4*	
ID1	Bathymetric Position Index	ID70	Mean of Residuals*	ID158	Representativeness*
ID2	Center vs. Neighbor variability*	ID178	Slope	ID188	Slope Variability
ID42	Easterness*	ID221	Value Range	ID227	Vector Ruggedness Index
ID67	Local Mean	ID31	Easterness	ID67	Local Mean
ID90	Northerness	ID90	Northerness	ID31	Easterness
ID166	Slope	ID190	Standard Deviation	ID90	Northerness

Scenario A: Each Selection used Alone (6 layers)

Scenario B: Each Selection used with Depth (7 layers)

Scenario C: Each Selection used with the Three Backscatter Derivatives (9 layers)

Selection D: Each Selection used with Depth and the Three Backscatter Derivatives (10 layers)

#### Unsupervised Classifications of Potential Habitat Types

Biophysical classifications of the area were performed to create benthoscape maps [10], which are produced by following a landscape style approach like when landscape features are delineated from terrestrial datasets. This top-down, unsupervised approach to habitat mapping is often used to map features that can only be resolved within the remotely sensed data, without attempting to delineate features beyond what the remote sensing techniques are capable of resolving. A total of 29 benthoscape maps were built using the Modified k-Means unsupervised classification tool in Whitebox GAT v.3.2 “Iguazu”. Algorithms such as k-means are commonly used in both terrestrial and marine ecological applications [5], but this particular algorithm is different from the regular k-means ones as it does not require a subjective input from the user to define the number of classes. The algorithm first segments the MBES derived data layers into a liberal, overestimated number of units, and then iteratively merges classes based on a pre-defined distance threshold between their cluster centres, eventually reaching an optimal, objective number of units. These units are then compared and subsequently recombined based on best match against independently classified *in situ* photographic data, classified into broad biophysical benthoscape classes. Using this approach, biophysical features can be delineated at a broader scale over the study area to generate a benthoscape map.

To assess the relative importance of the different environmental variables and the consequences of using different input variables in habitat mapping, four scenarios were tested with each of the seven selections, resulting in 28 habitat maps. Maps were first created using each selection alone (six input layers), then adding the bathymetry (seven layers), the three backscatter derivatives (nine layers), and finally both the bathymetry and the backscatter derivatives (ten layers). In order to quantify the relative influence of terrain morphology in potential habitat characterization of German Bank, an additional habitat map was produced using only the backscatter derivatives and the bathymetry (four layers, not accounting for terrain morphology).

Following the method outlined in Brown *et al*. [8], the resulting clusters for each classification were spatially compared to the 3,190 photographs of the seabed. Clusters corresponding to the same habitat types were grouped together and mapped as the corresponding habitat types. Confusion matrices, summarizing agreement and disagreement between the ground-truth data and the results from the classified bottom types [11], were built to compute the overall accuracy and kappa coefficient of agreement of each habitat map. The two measures are commonly used in ecology [12] and in remote sensing [11,13]. The success of the discrimination of each individual bottom type by the 29 classifications was assessed using the producer’s accuracy [11], and a spatial comparison of the outputs was made to assess the amplitude of change caused by selecting different variables. This was quantified using the percentage of pixels that were classified as the same bottom type by different classifications.

#### Supervised Classifications of Sea Scallop Habitats

In addition to the unsupervised classifications, a bottom-up supervised approach to habitat mapping was used in which the *in situ* data were used to segment the environmental data to predict sea scallop (*Placopecten magellanicus*) habitat on German Bank. Maximum entropy (MaxEnt) [14,15], a presence-background method, was used to perform these supervised classifications of scallops habitat. We recognize that there are benefits and drawbacks associated with all modelling techniques and that there is still a debate surrounding which one works best [8]. For the purpose of this study, a technique that could be kept consistent across the methodology was required in order to enable comparisons of outcomes. While any technique could have been used, MaxEnt was selected because it was shown to perform better than other species distribution models (SDM) in both terrestrial [16] and marine realms [17]. Following the method of Brown *et al*. [8], the classifier was run in the MaxEnt software v.3.3.3k with the default settings, except that the number of background points was increased to 50,000 to account for background conditions in full measure in such a large area. The 3,813 scallop observations selected by Brown *et al*. [8] were used to train the model, while the remaining 1,003 observations were kept for validation. A total of 29 MaxEnt models were run: for each of the seven selections, four models were run according to the scenarios previously mentioned resulting in 28 models, and one model was run without terrain attributes.

The MaxEnt software was also used to perform jackknife tests and to calculate the area under the curve (AUC) derived from threshold independent receiver operating characteristic (ROC) curves; the former quantify the percentage contribution of each input variable to the models while the latter serves to assess the performance of SDMs [16]. We acknowledge that there is currently a debate in the literature surrounding the use of AUC as a measure of model evaluation [18]; some authors argue that AUC can be inappropriate when different modeling techniques are used [19] or if two different species or areas are compared [20]. However, AUC often performs better than other measures [21,22] and is appropriate when the species, study area, and the training and test samples are the same across the compared models [23, 24], like in the current study.

Model outputs were evaluated in terms of their statistical fit to the validation data (AUC_Test_) [25]. A 95% confidence interval based on the standard deviate (1.96 standard deviations of the AUC_Test_ value) was used to identify the significant differences in performances [26]. The goodness-of-fit of the models to the training data (AUC_Train_) was used to assess models’ generalizability (*i*.*e*. transportability, transferability). Generalizability is described by Vaughan & Ormerod (p.720 [22]) as “a basic requirement for predictive models” that describes the ability of a model to produce accurate predictions with data other than the training dataset. Generalizability was measured using the difference (AUC_Diff_) between AUC_Train_ and AUC_Test_ [27]. A model that over-fits the training data will have a high AUC_Train_ but a low AUC_Test_ as it performs poorly on the test dataset, thus resulting in a high AUC_Diff_. Such a model is too specific to the training data and less generalizable. A diagnostic of the input variables contribution to the different models was also performed based on the results from the jackknife procedure, in order to identify the loss or gain in explanatory power as each variable is removed from the models or used alone [28]. Finally, a spatial comparison of the models was performed to evaluate the consequences of variable selection on the model outputs.

## Results

### Unsupervised Classifications

#### Performance of Classifications

**Fig 2:**
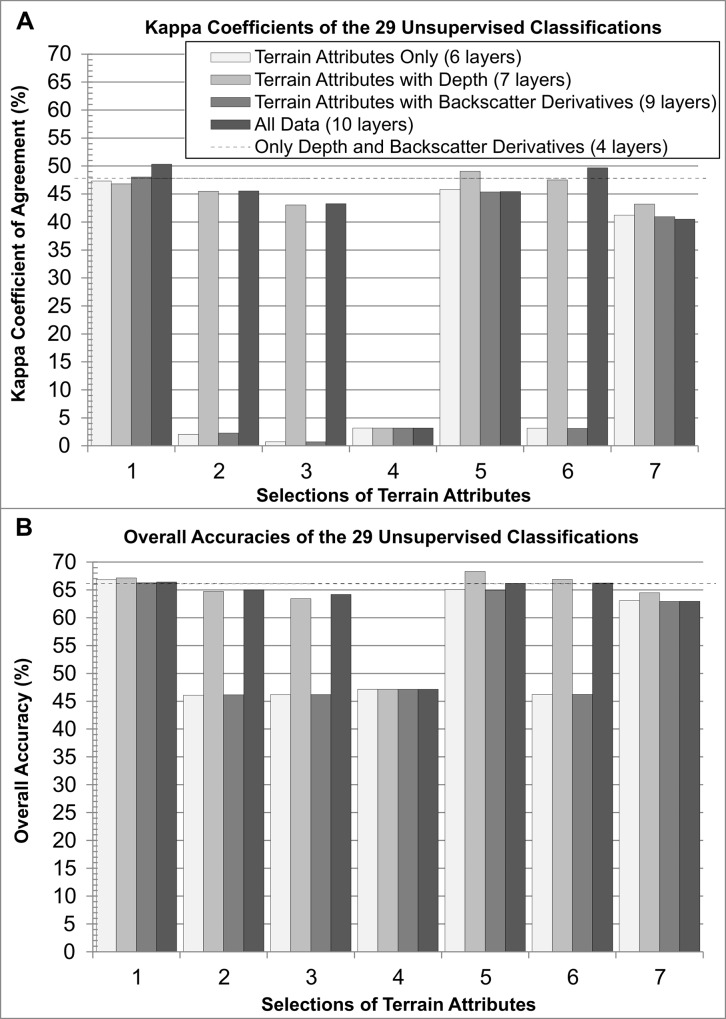
**Map accuracies measured with (A) a kappa coefficient of agreement and (B) the overall accuracy.**

The overall accuracies and kappa coefficients of the 29 habitat maps are presented in Fig 2. Selection 1 (*i*.*e*. the proposed attribute selection) outperformed the others with the highest overall accuracy and kappa coefficient in three of the four scenarios. The highest kappa coefficient was obtained when combining Selection 1 with bathymetry and the backscatter derivatives. The highest overall accuracy, 68.3%, was reached when combining Selection 5 with bathymetry (Fig 2B). Selection 1 combined with bathymetry had the second highest overall accuracy (67.1%). Selections with only three attributes from Selection 1 (*i*.*e*. Selections 5, 6 and 7) usually outperformed their related selection with none of the proposed attribute (*i*.*e*. Selections 2, 3 and 4). Selection 4 resulted in poor classifications, and Selections 2, 3 and 6 performed generally poorly except when bathymetry was added. Compared to the classification that only used bathymetry and the backscatter derivatives (*i*.*e*. no topography), eight classifications had a higher overall accuracy: the four classifications that used Selection 1 as input, Selections 5 and 6 combined with bathymetry, and Selections 5 and 6 combined with both bathymetry and the backscatter derivatives. In terms of kappa coefficients, only four classifications performed better than the one with no topography: Selection 1 with the backscatter derivatives, Selection 1 with both bathymetry and the backscatter derivatives, Selection 5 with bathymetry, and Selection 6 with both bathymetry and the backscatter derivatives.

Differences up to 45.5% were observed between the overall accuracy values and the kappa coefficients for a same selection and scenario. Differences were substantial with an average of 28.5% and a standard deviation of 11.9%. The average difference between the two measures of accuracy for the four maps using Selection 1 was the lowest, followed by the average difference for the four maps of Selections 5, 7, 6, 2, 3 and 4.

#### Discrimination of Benthoscape Classes

**Fig 3:**
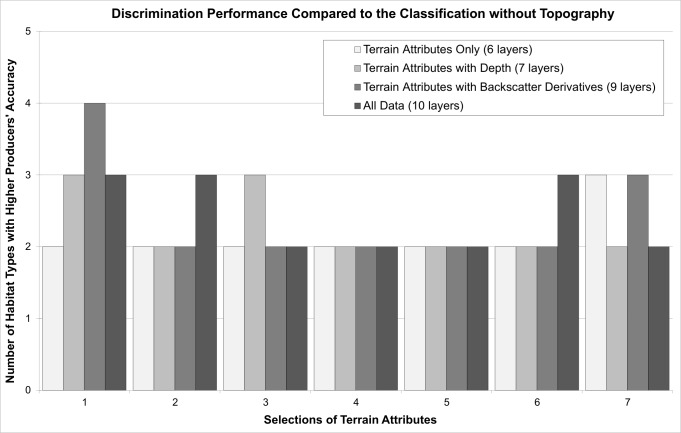
Comparison of the discrimination ability of the computed classifications with that of the classification computed using only bathymetry and the backscatter derivatives, based on the number of bottom types (maximum possible of 5) that were better discriminated.

Selection 1 performed on average better than the others when discriminating between the five bottom types (Fig 3). When looking at the individual habitat types, 25 of the 28 other classifications discriminated glacial till better than the classification with only bathymetry and the backscatter derivatives (producer’s accuracy of 77.1%), indicating that terrain morphology is not a good surrogate of the presence of glacial till. The “silt and mud” class seemed driven primarily by bathymetry and sediment properties (*i*.*e*. backscatter derivatives), with a producer’s accuracy of 87.4% for the classification that did not account for terrain morphology. Only two of the 28 remaining classifications discriminated that habitat type better, although several other classifications were very close to achieving that accuracy. Reefs were generally poorly discriminated. The classification with no topography reached a producer’s accuracy of 19.8%, and only six of the remaining classifications performed better, including three of the classifications using Selection 1. Rippled silt seemed to be better explained by the bathymetry and the backscatter derivatives, with the corresponding classification reaching an accuracy of 57.6%. Only four other classifications did better, including two classifications that included Selection 1. Finally, rippled sand was very poorly discriminated by all the classifications, which may be due to its small sample size (only 49 photographs).

In terms of mean producer’s accuracy for the five habitat types, only three classifications did better than the one with no topography (48.4%): Selection 1 with the backscatter derivatives (48.5%), Selection 1 with bathymetry and the backscatter derivatives (51.6%), and Selection 6 with bathymetry and the backscatter derivatives (51.3%). When averaging the mean producer’s accuracies from the four scenarios for each selection, Selection 1 ranked first, followed by Selections 5, 7, 6, 3, 2 and 4.

#### Spatial Variations of Outputs from Different Selections

The most accurate map according to the kappa coefficients of agreement was made from Selection 1 combined with bathymetry and the backscatter derivatives. The spatial similarity indices of that map with the other habitat maps built with ten layers are presented in Table 2. Compared to Selection 1, Selection 6 produced the most spatially similar map with 90.0% similarity. Selection 4 is the least similar with only about 41.7% identically classified pixels. The other maps were between 73.3% and 79.4% similar to the map with Selection 1, except for the map with no topography (*i*.*e*. only bathymetry and the backscatter derivatives) with 82%.

**Table 2:** Spatial similarity of the habitat maps and SDMs generated from Selections 2 to 7, compared to the map and model built from Selection 1. A similarity of 90% indicates that 90% of the pixels were classified as the same habitat type in the two compared maps, or that 90% of the pixels were within ±5% of probability distribution in the two compared models.

	Spatial Similarity with Selection 1 (%)	
	Scenario with 10 Layers	
	*Unsupervised Classifications*	*Supervised Classifications (within ±5% probability)*
Selection 2	73.3	72.9
Selection 3	77.0	64.6
Selection 4	41.7	65.3
Selection 5	79.4	81.4
Selection 6	90.0	71.5
Selection 7	78.4	70.9
No topography	82.1	66.9

### Supervised Classifications

#### Predictive Capacity and Robustness

**Fig 4:**
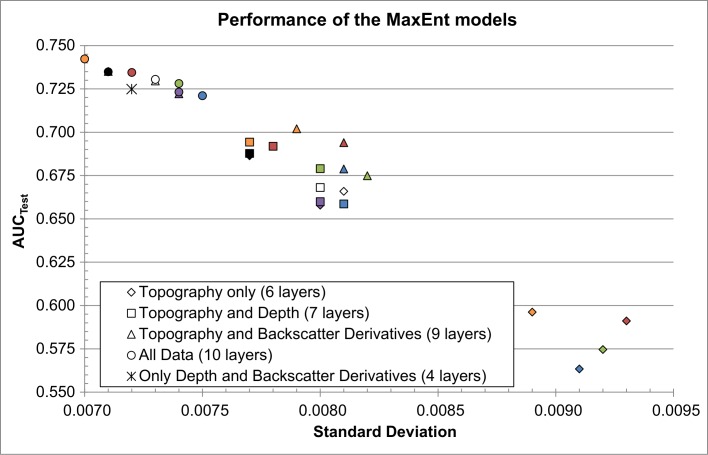
Performance and robustness of the 29 MaxEnt models. Models in the top-left corner of the graph performed better and are more robust. Colour legend: Selection 1 (black), Selection 2 (blue), Selection 3 (red), Selection 4 (green), Selection 5 (purple), Selection 6 (orange), Selection 7 (white).

Fig 4 shows the performance of the 29 MaxEnt models. All models performed significantly better than random (*i*.*e*. AUC_Test_ ± 95% confidence interval > 0.500). Models with higher AUC_Test_ and lower standard deviations are more robust and present the highest predictive capacity [29]. In general, adding bathymetry, the backscatter derivatives, or all of them to the terrain attributes improved the models predictive capacity. However, Selection 1 and other selections that include terrain attributes from Selection 1 did not always follow that trend. For instance, Selection 1 used alone (only six terrain attributes; black diamond in Fig 4) performed better than other selections combined with bathymetry or the backscatter derivatives (*e*.*g*. blue and green squares and triangles in Fig 4). Selection 1 combined with the backscatter derivatives (black triangles in Fig 4) performed better than other selections that were combined with both bathymetry and the backscatter derivatives (*i*.*e*. most circles in Fig 4).

In the scenario where only terrain attributes are used (diamonds in Fig 4), Selection 1 performed the best, followed by the three selections that include three terrain attributes from Selection 1 (Selections 5, 6, and 7). The same pattern was observed when combining the selections with the three backscatter derivatives (triangles in Fig 4). A different pattern arose when adding bathymetry to the selections, one in which Selection 1 performed second best behind Selection 6. However, the 95% confidence intervals measured around the AUC values show that the difference in performances between Selection 1 and 6 are not significant for the two scenarios where Selection 6 performed better than Selection 1.

#### Generalizability

**Fig 5:**
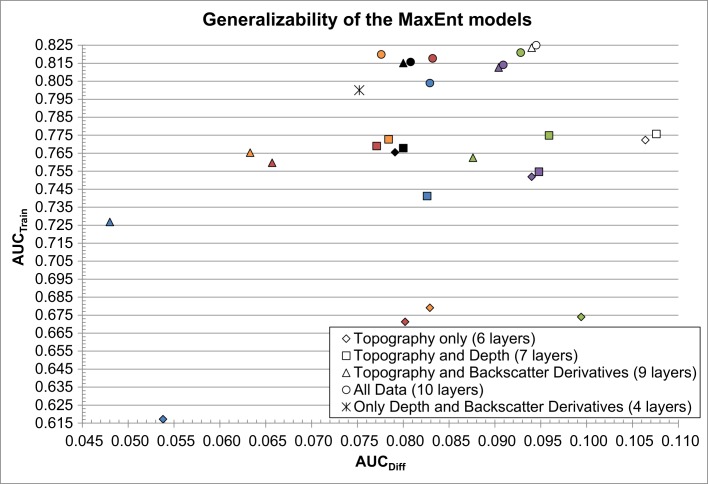
Generalizability of the 29 MaxEnt models. Models closer to the top-left corner are more generalizable as they performed well on the training data and replicated well to the validation data. See Fig 4 for colour legend.

Fig 5 shows the generalizability of the 29 SDMs. Models with higher AUC_Train_ fitted better the training data while models with lower AUC_Diff_ predicted more efficiently the validation data. Models with high AUC_Train_ and low AUC_Diff_ are therefore the most generalizable, as they do not over-fit the training data [29]. Fig 5 shows that the models that included bathymetry (scenarios with seven and ten layers; squares and circles in Fig 5) are more similar than the other models, especially for the models that combined ten input layers.

Models that used only terrain attributes or combined them with backscatter derivatives (diamonds or triangles in Fig 5) showed similar patterns, where the best models in terms of AUC_Train_ also had a higher AUC_Diff_, an indication that the best models were also the ones that over-fitted the data the most. In those two scenarios, Selection 1 clearly stands out as a good trade-off between predictive ability and over-fitting of data, making it the most likely to be generalizable and to perform well. When considering bathymetry (squares and circles in Fig 5), a similar pattern emerged whether or not the backscatter derivatives were added: Selections 1, 3 and 6 stand out as being more generalizable. Selections 7 and 4 have the highest AUC_Train_, but also the highest AUC_Diff_, therefore having a tendency to over-fit the training data.

#### Variables Contribution

**Fig 6:**
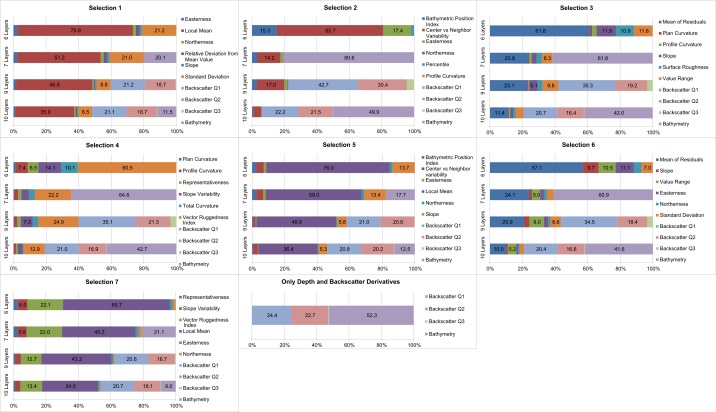
Percentage of variable contribution for the 29 MaxEnt models. Only contributions greater than 5% are labeled.

The percentage of contribution of each variable used as input in the 29 models can be found in Fig 6. When used, bathymetry and two of the backscatter derivatives (Q1 and Q2) contributed the most to the models, with a respective average of 39.2%, 25.4% and 19.6% for the 15 models that used them. Bathymetry contributed less to the models that include local mean as input, resulting from the high collinearity between these two variables; when two variables are correlated, MaxEnt is known to assign a more important percentage contribution to one of the two and a lower one to the other [28]. Consequently, local mean is a surrogate of bathymetry and appears as an important variable, with an average contribution of 51.2% for the 12 models that include it. In general, measures of rugosity like standard deviation and vector ruggedness measure also contributed to the models.

The analysis of changes in model gain based on the jackknife procedure described the impact on model gain of removing each variable from the models, in addition to provide what would be the model gain if each variable would be used alone. This analysis provided additional information on the variables contribution and the performance of models. In MaxEnt, a variable with a high gain when used alone in a model contributes useful information to the model [28]. On the other hand, a variable that contributes unique information to a model makes the gain decrease when it is excluded from the model [28]. In this study, all variables in all models provided unique information in training the models, except for the four models that included Selection 2. In terms of transferability of this uniqueness to the training data (*i*.*e*. if the variables still provide unique information when applied to the validation data), Selection 1 performed better than the others in three of the four scenarios. It only failed to outperform the other selections when ten layers were used, likely due to spatial correlations between local mean and bathymetry, and slope and local standard deviation. Regarding usefulness, Selection 1 generally did not provide as many useful variables to the models being trained as the other selections. However, these useful variables were generally also useful for the validation data, thus transferable, which was not the case for the other selections. For instance, Selection 1 in combination with bathymetry and the backscatter derivatives had six variables providing useful information to the trained model, and these six variables were all useful for the validation data, an indication of robustness and generalizability. Finally, only two models would not have reached a higher AUC_Test_ if any one of their inputs were removed: Selection 1 combined with the backscatter derivatives and Selection 1 combined with bathymetry.

#### Spatial Variations of Predictions from Different Selections

The model computed from the combination of Selection 1 with bathymetry and the backscatter derivatives showed the best trade-off between robustness, uniqueness and generalizability. It was therefore used as a reference to spatially compare the outputs of comparable models, *i*.*e*. those computed with ten layers (Table 2). The most similar model to the reference one, based on a ±5% margin in probability distribution, was the model computed with Selection 5 (81.4% similar). The lowest similarity was 64.6% (Selection 3). In average, the six other models were 71.1% similar to the one made from Selection 1. The map produced without terrain morphology had a spatial similarity index of 66.9% with the map from Selection 1 combined with bathymetry and the backscatter derivatives.

## Discussion

### Selections of Terrain Attributes

Results suggest that Selection 1 of terrain attributes, which corresponds to the combination of a measure of relative position, a measure of rugosity, two measures of aspect (easterness and northerness), topographic mean and slope, is more appropriate than the other selections tested. First, the proposed selection of terrain attributes performed better than the other selections tested, both in the application of top-down and bottom-up approaches to habitat mapping: they generally (1) produced more accurate habitat maps, (2) better discriminated individual habitat types, (3) produced SDMs with higher AUC values, (4) produced more robust and generalizable SDMs, (5) provided SDMs with the most variables carrying unique information, and (6) had the highest number of variables carrying useful information that replicated well to the validation data. Using real data, these results confirm that the six recommended terrain attributes best describe the topographic structure of the terrain by capturing different and unique characteristics of the terrain. Results also indicate robustness and generalizability of the proposed framework. Many aspects of this study highlighted better performances of Selection 1 compared to Selections 2, 3 and 4, thus confirming the limited ability of these three selections to adequately and fully describe terrain geomorphology.

The findings from this study, utilizing MBES-derived surfaces from German Bank, support many of the findings presented by Lecours *et al*. [4] based on terrain attributes generated from artificial surfaces. Their proposed operational framework was based on two literature-grounded assumptions: fractal-based surfaces created with spectral synthesis are appropriate representations of natural surfaces [30,31], and the scale-invariance property of fractals allows results to be generalized to other spatial scales (*i*.*e*. different resolution and/or extent) [32,33]. Artificial surfaces proved their value in ecology [34] and geomorphometry [3]. DTMs of real terrains are actually geographic “representations” of real terrains, thus in theory no different than DTMs representing artificial terrains with characteristics found in real terrains. However, a number of authors argue that fractal-based surfaces should be limited to the development of null hypotheses [35,36]. The debate is still unsettled; while some claim that “it is heuristically clear that seafloor or landscape topography is best described by fractal geometry” (p.981 [37]), others prefer to argue that despite demonstrating fractal-like properties [38], real terrains are not perfectly fractal [39]. Without necessarily contributing to this debate, the current study confirmed that results gained from the artificial fractal surfaces in Lecours *et al*. [4] hold when using a DTM representation of a real terrain (*i*.*e*. German Bank) at another spatial scale (*i*.*e*. an extent of 3650 km^2^ represented at 50 m resolution). Consequently, it confirmed the appropriateness of the proposed framework for selecting terrain attributes and its application to any terrestrial and marine ecological application, regardless of the scale of the environmental data.

### Terrain Morphology as an Environmental Factor

Results of both types of classifications indicate that bathymetry and substrate characteristics (for which the backscatter derivatives were a proxy) had a positive, and sometimes more important impact on the performance of the classifications than terrain morphology (quantified through terrain attributes); adding bathymetry and the backscatter derivatives to terrain attribute variables often increased map accuracy for both benthoscape and sea scallop suitability distributions on German Bank. For the unsupervised classifications, only two of the five bottom types (glacial till and reefs) seemed to be driven to a certain level by local geomorphology. In addition, reefs and rippled silt were poorly discriminated by a majority of classifications, likely because their distribution is influenced by other environmental factors or that the variables tested were measured and analyzed at a scale that did not match the scale of the relevant geomorphological features [6]. In agreement with results from Brown *et al*. [8], the MaxEnt analysis showed that bathymetry, sediment properties and rugosity are important variables in predicting sea scallops distribution, but that aspect, slope and relative position are not. Only four SDMs out of 28 performed better than the model with no topography (only bathymetry and the three backscatter derivatives).

Other variables (*e*.*g*. physical, oceanographic, ecological) may drive particular species or assemblage distributions more than terrain geomorphology. However, they were not used in this study as they were not available at the same spatial scale as the MBES data. When including more variables, users need to keep in mind that covariation may influence models like MaxEnt. If an oceanographic variable is correlated with a terrain characteristic, the user needs to keep only one of them. This is also true of the proposed selection of terrain attributes; as demonstrated in Lecours *et al*. [4] and confirmed in the current study, each of the six proposed terrain attributes captures a unique characteristic of the terrain, but some of these characteristics may be spatially correlated in a certain area.

The framework for selecting terrain attributes for ecological studies proposed by Lecours *et al*. [4], and supported by the findings of this study, aims at helping the end-users select a robust combination of terrain attributes that best captures the different characteristics of terrain geomorphology. The recommended selection of six terrain attributes serves as a guide as to which set of attributes should be tested in order to achieve the best outcome. It provides end-users with an optimal set of attributes, from which a subset combined with other environmental variables can result in a high accuracy map or model output. The best results will not necessarily come from the use of all six terrain attributes, but may only come from some of them. For instance, if particular terrain characteristics have no ecological meaning in an application, using the terrain attributes that capture these characteristics will not yield the best outcome. It is therefore highly site and case specific as to which variables should be included [6]. Nonetheless, the recommended approach provides the optimal starting point from which terrain attributes can be selected.

### Consequences of Variable Selection

Results highlight the importance of appropriately selecting input variables in both unsupervised and supervised classifications, and consequently the inappropriateness of making such selection arbitrarily. For instance, the benthoscape map generated from the combination of Selection 1 with bathymetry and the backscatter derivatives yielded an overall accuracy and a kappa coefficient of agreement that are respectively only 0.2% and 0.6% different than the map built from Selection 6, bathymetry and the backscatter derivatives. It would be quite intuitive to interpret the difference in map outputs as insignificant based only on these measures of accuracy. However, 10.0% of the study area was classified differently by these two classifications, an area corresponding to about 362 km^2^. In addition, the differences occurred in all regions of the study area and across all the habitat types. In the worst case scenario (*i*.*e*. the difference between Selection 4 and Selection 1, Table 2), the total area that was mapped differently covers over 2,115 km^2^. The results of this study indicate that a subjective selection of terrain attributes could potentially provide a map that is in average 26.7% different in terms of the location and boundaries of benthoscape classes, which has serious implications for ecological applications that use these maps and models for decision-making.

### Comparisons with Other Studies: Terrestrial and Marine

Many different terrain attribute selections have been used in terrestrial and marine ecology ([1,7]; references therein). In a meta-analysis of ecological studies using geomorphometry, Bouchet *et al*. [1] found that about a third of the studies only used one terrain attribute and that very few authors used more than four. While focusing on forest ecosystems, Sharaya & Sharyi [40] wrote that in general, one to three basic terrain attributes are used to study landscape phenomena and that the “insufficient representativeness” (*ibid*, p. 2) of terrain attributes makes for an inefficient use of topography as a variable in ecology. In a management context and using the same dataset as in the current study, Brown *et al*. [8] selected six terrain attributes based on previous use in marine ecology studies and “iterative testing of a large number of different layers by the authors” (*ibid*, p. 3). This relatively subjective way of selecting terrain attributes is the most common one in ecology. However, it provides many significant and valid insights for many applications; most of the common terrain attributes found in the ecological literature (*e*.*g*. local mean, slope, aspect) [1] are part of our proposed selection, or are related to one of the proposed attributes. For instance, different types of curvature are commonly used, which Lecours *et al*. [4] found to be correlated to the recommended relative deviation from mean value, although more ambiguously defined and thus not included in the recommended selection. Despite using a subjective selection of terrain attributes, Brown *et al*. [8] yielded valid results. Their MaxEnt model had a high predictive capacity, although it had some level of over-fitting and was less robust than some of the best models of the current study. If implemented in the current study using the same method, an unsupervised classification made from their selection of variables would rank amongst the best benthoscape maps and be 90.8% similar to the map built with Selection 1 and the four other environmental variables. This demonstrates that despite potentially resulting in huge differences (*c*.*f*. “Consequences of Variable Selection” above), subjective selection of terrain attributes can sometimes produce relevant and valid results. As a matter of fact, the model by Brown *et al*. [8] has been used in subsequent studies and to inform fisheries stock assessment process and management [41,42,43,44].

Finally, the observed differences between the overall accuracy measures and the kappa coefficients of agreement confirm that the overall accuracy might be a poor guide of the value of a classification, something that has been already argued in the literature [45,46]. Based on our results, we would recommend the kappa coefficient as a more appropriate measure than the overall accuracy for ecological mapping, but as recently highlighted by Diesing *et al*. [5], there is a need to move towards spatial representations of accuracy.

## Conclusion

Selecting the most appropriate environmental variables to use in a specific study can be very challenging. This study demonstrated the importance of carefully selecting variables for ecological work; maps and models that perform similarly can still produce very different spatial outcomes, which can have important implications when these maps and models are used in decision-making for conservation and management. Using two different approaches to habitat mapping, this paper also confirmed that the selection of terrain attributes recommended in Lecours et al. [4] performs better than other selections, thus serving as a guide to make better use of geomorphometry in ecology. Results also showed that while this selection of terrain attributes ensures that most of the local topographic structure is captured when performing terrestrial or marine ecological studies, and while terrain morphology can help improve maps and models, it is not always the most important environmental factor for all ecological applications. The relationship between terrain morphology and ecological phenomena is species, area and scale-dependent [6]. The use of the proposed selection of terrain attributes, in combination with other environmental variables (*e*.*g*. precipitations, climate, currents), will help ecologists produce more robust analyses and generate maps and models with a higher degree of confidence. In order to get the best representation of the environment as possible and to best inform policy, conservation and management efforts, we recommend (1) that stakeholders prepare more than a single map using different combinations of environmental variables, and (2) that they select the best outcome based on map accuracy or model performance quantification.

## Supporting Information

S1 Appendix Additional information on the selections of variables. (PDF)
