# Supplementary material for: Comparing Selections of Environmental Variables for Ecological Studies: A Focus on Terrain Attributes
Source: PLoS One. 2016 Dec 21;11(12):e0167128. doi: 10.1371/journal.pone.0167128 (PMC5176161; doi:10.1371/journal.pone.0167128)
Supplement: S1 Appendix — (PDF) [file pone.0167128.s001.pdf]

Appendix S1: Additional information on the selections of variables

The seven combinations of terrain attributes that were compared were built based on results from a related study [1], which aimed at (1) identifying terrain attributes that represent unique terrain characteristics and (2) suggesting a combination of these terrain attributes that would be optimal in capturing as much of the terrain structure as possible (*i.e.* in summarizing a surface). A total of 230 terrain attributes computed from 11 different software packages were derived from nine different terrain surfaces. To reach the objectives, three multivariate statistical methods that explored both linear and non-linear relationships amongst terrain attributes were combined: Principal Component Analysis (PCA), Variable Inflation Factor (VIF), and Mutual Information (MI).

The first statistical analysis performed on the datasets of terrain attributes for each surface was an iterative PCA. Before proceeding with the PCA analyses, the data were cleaned by looking at the cardinality of the different terrain attributes. Cardinality is the number of different values for a variable. Terrain attributes with low cardinality were not used as input in PCA analyses as such variables are known to complicate PCA solution and only account for a negligible amount of the total variance. *Selection 2* was built from variables that were removed at this step.

To enable generalization of results, the PCA solutions needed to reach a simple structure. A simple structure is reached when most components have “marker” variables, *i.e.* those that load strongly on only one component and thus contribute to explain unique variation within the dataset. Variables that load strongly on more than one component are called “complex” variables and are considered redundant. *Selection 1* and *Selection 4* were built from marker terrain attribute variables, while *Selection 3* was built from complex variables. The difference between *Selection 1* and *Selection 4* is linked to where the terrain attribute variables loaded in the PCA solution. The terrain attributes from *Selection 1* loaded on the first few components, which are the strong ones that explain greater amounts of variation in the dataset. Those from *Selection 4* loaded strongly on further components, which account for smaller amount of variation in the dataset.

Selection 5, *Selection 6* and *Selection 7* were built on the assumption that variables from *Selection 1* were indeed optimal. Each of those selections were built from the combination of three terrain attributes from *Selection 1* and three terrain attributes from respectively *Selection 2*, *Selection 3* and *Selection 4*.

In addition to PCA, two independent stepwise measures of covariation (VIF and MI) were used on the terrain attribute variables. These two measures ranked the variables from least covarying to most covarying, and results were used to confirm PCA results and explore the uniqueness of the variables that did not load on any component, had a very low amount of variance accounted for by the sum of the components, or were not considered in the PCA because of their low cardinality. Variables with one of these three characteristics and that

were ranked amongst the least covarying variables were identified as potentially important and requiring further investigation. A particular focus was thus given to these terrain attributes when building the different selections (identified by a * in Table 1 of the main text).

Table A.1: List of terrain attributes with software, algorithms and references. The ID refers to [1].

ID	Attributes Names' in Software	Software	Algorithms/Methods/References
1	Bathymetric Position Index	ArcGIS 10.2.2 and Benthic Terrain Modeler 3.0 Release Candidate 3	[2]
2	Center versus Neighbors Variability	Idrisi Selva 17.0	Not Specified
31	Easterness	ArcGis 10.2.2 and Python 2.7.8	[3]
42	Easterness	SAGA GIS 2.0.8	Maximum Slope [4]
67	Mean	ArcGis 10.2.2 and Python 2.7.8	Not Specified
70	Mean of Residuals	Landserf 2.3	Not Specified
90	Northerness	ArcGis 10.2.2 and Python 2.7.8	[3]
101	Northerness	SAGA GIS 2.0.8	Maximum Slope [4]
111	Percentile	SAGA GIS 2.0.8	Exponential [5]
116	Plan Curvature	ArcGis 10.2.2 and DEM Surface Tools for ArcGIS 10 (v.2.1.399)	[6]
132	Plan Curvature	Whitebox GAT 3.2.1 Iguazu	[7]
136	Profile Curvature	ArcGis 10.2.2 and DEM Surface Tools for ArcGIS 10 (v.2.1.399)	[6]
143	Profile Curvature	SAGA GIS 2.0.8	Least Squares Fitted Plane [8,9]
153	Profile Curvature	Whitebox GAT 3.2.1 Iguazu	[7]
157	Relative deviation from mean	ArcGis 10.2.2 and Python 2.7.8	Not Specified
158	Representativeness	SAGA GIS 2.0.8	[10]
166	Slope	ArcGis 10.2.2 and Python 2.7.8	[8]
178	Slope	SAGA GIS 2.0.8	Maximum Slope [4]
188	Slope Variability	ArcGis 10.2.2 and Python 2.7.8	[11]
190	Standard Deviation	ArcGis 10.2.2 and Python 2.7.8	[12]
201	Surface Roughness Index	ArcGis 10.2.2 and Python 2.7.8	[13]
219	Total Curvature	ArcGis 10.2.2 and DEM Surface Tools for ArcGIS 10 (v.2.1.399)	[6]
221	Value Range	SAGA GIS 2.0.8	Exponential [5]
227	Vector Ruggedness Measure	SAGA GIS 2.0.8	Exponential [5]

Literature Cited

[1] Lecours V, Devillers R, Simms AE, Lucieer VL, Brown CJ (under review). Towards a framework for terrain attribute selection in environmental studies.

[2] Wright DJ, Pendleton M, Boulware J, Walbridge S, Gerlt B, Eslinger D, Sampson D, Huntley E. ArcGIS Benthic Terrain Modeler (BTM), v. 3.0, Environmental Systems Research Institute, NOAA Coastal Services Center, Massachusetts Office of Coastal Zone Management. 2012. Available from http://esriurl.com/5754.

[3] Zevenbergen LW, Thorne CR. Quantitative analysis of land surface topography. Earth Surf Proc Land, 1997;12:47-56.

[4] Travis MR, Elsner GH, Iverson WD, Johnson CG. VIEWIT: computation of seen areas, slope, and aspect for land-use planning. 1975; USDA F.S. General Technical Report PSW-11/1975, 70p.

[5] Wilson JP, Gallant JC. Terrain analysis: principles and applications. Wiley, Hoboken. 2000.

[6] Evans IS. An integrated system of terrain analysis and slope mapping. Final report on grant DA-ERO-591-73-G0040, University of Durham, England. 1979.

[7] Gallant JC, Wilson JP. Primary topographic attributes. Terrain Analysis: Principles and applications, *in* Wilson JP, Gallant JC (eds). Wiley, Hoboken. 2000;51-86.

[8] Horn BKP. Hill shading and the reflectance map. P IEEE. 1981;69:14-47.

[9] Costa-Cabral M, Burgess SJ. Digital Elevation Model Networks (DEMON): a model of flow over hillslopes for computation of contributing and dispersal areas. Water Resour Res. 1996;30:1681-1692.

[10] Boehner J, Koethe R, Trachinow C. Weiterentwicklung der automatischen Reliefanalyse auf der Basis von Digitalen Gelandemodellen. Gott Geogr Abhand. 1997;100:3-21.

[11] Ruszkiczay-Rüdiger Z, Fodor L, Horváth E. Discrimination of fluvial, eolian and neotectonic features in a low hilly landscape: a DEM-based morphotectonic analysis in the Central Pannonian Basin, Hungary. Geomorphology. 2009;104:203-217.

[12] Ascione A, Cinque A, Miccadei E, Villani F, Berti C. The Plio-Quaternary uplift of the Apennine chain: new data from the analysis of topography and river valleys in Central Italy. Geomorphology. 2008;102:105-118.

[13] Hobson RD. Surface roughness in topography: quantitative approach, *in* Chorley RJ (ed.) Spatial Analysis in Geomorphology, Harper and Row, New York. 1972.
